# Influence of Endogenous Factors of Food Matrices on Avidin—Biotin Immunoassays for the Detection of Bacitracin and Colistin in Food

**DOI:** 10.3390/foods11020219

**Published:** 2022-01-13

**Authors:** Maksim A. Burkin, Inna A. Galvidis, Sergei A. Eremin

**Affiliations:** 1 Immunology Department, I. Mechnikov Research Institute for Vaccines and Sera, 105064 Moscow, Russia; galvidis@yandex.ru; 2 Faculty of Chemistry, M. V. Lomonosov Moscow State University, Leninsky Gory, 1, 119991 Moscow, Russia

**Keywords:** bacitracin, colistin, biotinylated hapten coating, immunoassay, matrix biotin and avidin interference, food contaminants

## Abstract

(Strept)avidin–biotin technology is frequently used in immunoassay systems to improve their analytical properties. It is known from clinical practice that many (strept)avidin–biotin-based tests provide false results when analyzing patient samples with a high content of endogenous biotin. No specific investigation has been carried out regarding possible interferences from avidin (AVI) and biotin (B_7_) contained in food matrices in (strept)avidin–biotin-based immunoanalytical systems for food safety. Two kinds of competitive ELISAs for bacitracin (BT) and colistin (COL) determination in food matrices were developed based on conventional hapten–protein coating conjugates and biotinylated BT and COL bound to immobilized streptavidin (SAV). Coating SAV–B_7_–BT and SAV–B_7_–COL complexes-based ELISAs provided 2- and 15-times better sensitivity in BT and COL determination, corresponding to 0.6 and 0.3 ng/mL, respectively. Simultaneously with the determination of the main analytes, these kinds of tests were used as competitive assays for the assessment of AVI or B_7_ content up to 10 and 1 ng/mL, respectively, in food matrices (egg, infant milk formulas enriched with B_7_, chicken and beef liver). Matrix-free experiments with AVI/B_7_-enriched solutions showed distortion of the standard curves, indicating that these ingredients interfere with the adequate quantification of analytes. Summarizing the experience of the present study, it is recommended to avoid immunoassays based on avidin–biotin interactions when analyzing biosamples containing these endogenous factors or enriched with B_7_.

## 1. Introduction

The interaction of (strept)avidin protein and biotin (vitamin B_7_) is widely used in various bioanalytical systems, in particular, in immunoassays [1]. Due to the high affinity (*K*_D_ ≈ 10^−15^ M) and binding valence between this protein (four binding sites) and the vitamin, they are successfully applied as immunoreagent labels to provide an additional functionality [2], for oriented immobilization/presentation [3], as well as to accelerate the interaction [4] or enhance the output signal in immunoassays of various designs [5,6]. In this regard, the involvement of the (strept)avidin–biotin system is one of the main strategies for increasing the sensitivity of immunoassay [7].

In a wide panel of commercial (strept)avidin–biotin-based immunoassay systems used in clinical practice for diagnostic purposes, it has been found that the presence of endogenous biotin in patients’ biofluids can interfere with the analysis, leading to false results and, consequently, to misdiagnosis and patient mismanagement [8,9]. However, with a dietary intake of about 35–70 µg B_7_/day, the blood B_7_ level in healthy subjects (0.12–0.36 nM) [10] has a negligible interfering effect on (strept)avidin–biotin assays. At the same time, excessive consumption as a result of therapy for a number of disorders (multiple sclerosis, phenylketonuria, biotinidase deficiency) or incorporation in “Hair, Skin, and Nails” cosmetic formulas may lead to μM B_7_ blood levels, which can significantly distort the results of (strept)avidin–biotin-based tests [9].

The (strept)avidin–biotin technology is equally popular in immunoassays for food safety control [11,12,13]. However, researchers have not yet been concerned about the possibility that avidin and biotin in the matrix could interfere with the detection of a wide range of analytes in food. Avidin (AVI) is a known protein component in egg white, whereas biotin is widely distributed in different natural foodstuffs. Foods relatively rich in biotin include egg yolk, liver, some vegetables [14], and the majority of biotin in meats and cereals appears to be protein-bound [10]. Thus, these endogenous components of the matrix, the content of which reaches ppb–ppm levels in many analyzed natural foods [15], can represent a serious obstacle in immunoassays based on (strept)avidin–biotin interaction.

In this regard, the objective of the present study was the assessment of the influence of these factors on newly developed ELISA systems based on the coating of biotinylated haptens for the determination of the antibiotics bacitracin and colistin in food matrices rich in AVI and B_7_ components.

**Figure 1:**
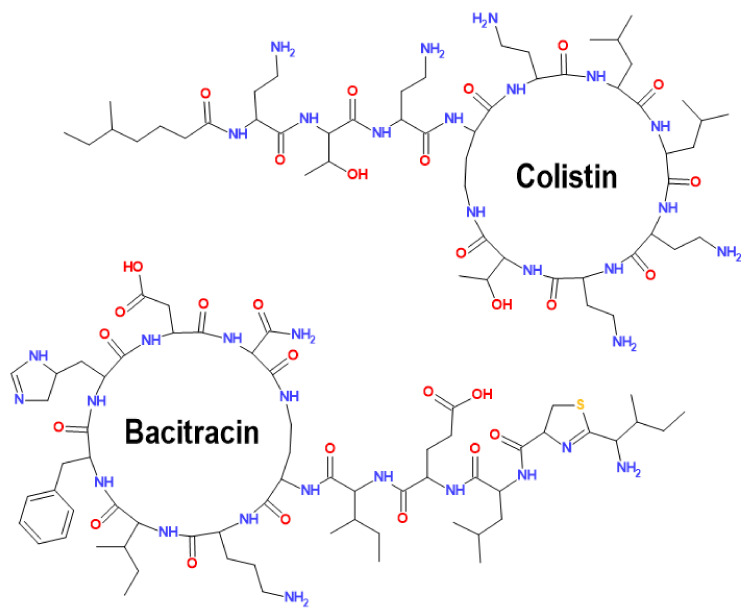
Structural formulas of the peptide antibiotics bacitracin and colistin.

Bacitracin (BT) [MW = 1422.7] and colistin (COL) [MW = 1155.4] are both cyclic peptide antibiotics (Figure 1) used in veterinary and human medicine. BT is produced by strains of *Bacillus licheniformis* and functions as an anti-Gram-positive agent, inhibiting bacterial cell wall biosynthesis [16]. Colistin is a product of the biosynthesis of *Paenibacillus polymyxa*, has a wide Gram-negative spectrum, and kills bacteria mainly through the disruption of bacterial outer membrane integrity, resulting from the binding with LPS [17]. 

To expand their antibacterial action, these antibiotics can be used together, potentiating each other’s activity [18]. Their use in farm animals and a proper withdrawal period should be controlled, so that the residual content of these antibiotics in agricultural products does not exceed the established acceptable threshold (Table 1).

Among the methods intended for the detection of food contamination with residues of the antibiotics BT and COL, immunochemical methods, in addition to physicochemical techniques [21], are described [22,23,24]. However, no method involves the (strept)avidin–biotin technology.

## 2. Materials and Methods

### 2.1. Chemicals

Colistin (COL) and polymyxin B (PMB) sulphates were obtained from AppliChem (Darmstadt, Germany). Bacitracin (BT), colistin methanesulphonate (CMS), 1-ethyl-3-(3-dimethylaminopropyl)carbodiimide (EDC), N-hydroxysuccinimide (NHS), biotin N-hydroxysuccinimide ester (NHS-B_7_), caprylic acid, bovine serum albumin (BSA), gelatin (GEL) horseradish peroxidase (HRP), and dimethylformamide (DMF) were purchased from Chimmed (Moscow, Russia). Avidins from *Streptomyces avidini* (streptavidin, SAV) and from egg white (AVI) were from Calbiochem (USA). Goat anti-rabbit IgG antibodies conjugated to HRP (GAR–HRP) were purchased from Imtek Ltd. (Moscow, Russia). Rabbit antisera against BSA–PMB and BSA–BT were prepared and described earlier [25,26], and IgG fractions were isolated using the caprylate–sulphate ammonium method according to a described procedure [27].

### 2.2. Preparation of Biotinylated Analytes

NHS-B_7_ was dissolved in anhydrous DMF (10 mg/mL), added dropwise to solutions of BT, COL, and PMB in 0.05 M carbonate–bicarbonate buffer (CBB, pH 9.6) at a molar ratio (1:1), and stirred 1 h at RT using a magnet stirrer. The biotinylated peptides were supplemented with equal volumes of glycerol and stored as 0.1 mM (B_7_) solutions at −20 °C until use.

### 2.3. Preparation of the Heterologous Coating Antigen GEL–BT(ae)

BT (1.42 mg, 1 µmol) in 0.284 mL DMF was added to EDC and NHS (2.5 eq of each) from 10 mg/mL solutions in DMF and stirred for 1.5 h. After activation of the carboxy groups, BT taken in 10- and 30-fold molar excesses over GEL (4 mg, 25 nmol) in CBB (pH 9.6) was added dropwise and stirred for 2 h at RT. Unconjugated hapten was removed by dialysis against water.

### 2.4. Preparation of the Heterologous Coating Antigen GEL(pi)–PMB

The glycoprotein GEL was oxidized with sodium periodate and conjugated to amines of PMB as a result of reductive amination, according to procedure described in [25]. GEL (4 mg, 25 nmol) and sodium periodate (1 mg, 5 μmol) in 0.5 mL of distilled water were mixed using a magnetic stirrer for 15 min. The oxidized GEL was dialyzed overnight against water at 4 °C and then added to the solution of PMB in CBB (pH 9.5) taken in 10- and 30-fold molar excess over the protein. After 2 h of stirring with a magnetic stirrer, 0.1 mL of sodium borohydride solution (2 mg/mL) was added to the reaction mixture, which was stirred for another 2 h. The resulting conjugates were dialyzed against water for 2 days at 4 °C.

### 2.5. Competitive ELISAs Based on the Coating Complex SAV–Biotinylated Hapten and Coated Conjugates

SAV was adsorbed on 96-well high-binding Costar plates (Corning, USA) from 1.0–3.0 µg/mL solutions in CBB at 4 °C. After overnight coating, the plates were washed three times with 0.15 M PBS (pH 7.2) containing 0.05% Tween 20 (PBST) and filled with biotinylated antibiotics (B_7_, 100–0.1 nM). The interaction of SAV and B_7_–hapten was carried out for 1 h at 37 °C. Alternatively, a one-step coating could be carried out using a mixture of SAV and B_7_–hapten previously prepared in PBS and coated overnight. The plates with the immobilized hapten were then washed and used for a conventional indirect competitive ELISA based on coated hapten–protein conjugates using specific rabbit anti-hapten antibodies and GAR–HRP [28]. Antibodies added to the wells in the working concentration in 1% BSA-PBST (0.1 mL) were incubated for 1 h on an ST-3 L plate shaker (ELMI Ltd., Latvia) at 25 °C. Antibody binding at zero analyte concentration (B_0_) was maximal and dose-dependently inhibited in the presence of free analyte (B, 0.01–1000 ng/mL). Bound antibodies were detected using GAR–HRP (1 h, 37 °C) and a TMB-containing substrate mixture (0.5 h, 25 °C). The enzymatic reaction was terminated by the addition of 2 M H_2_SO_4_, and the absorbance was read at 450 nm using a LisaScan spectrophotometer (Erba Mannheim, Czech Republic). The standard curves were plotted as ‘relative antibody binding B/B_0,_ % vs. analyte concentration’ using GraphPad Prism 8 Software and served for the measurement of antibiotics in food matrices. The analyte concentrations (IC_50_) causing 50% inhibition of antibody binding served as assay sensitivity values as well as to determine the cross-reactivity (CR) of analogs according to the equation: CR = IC_50 MAIN ANALYTE_/IC_50 ANALOGUE_ × 100%.

### 2.6. Assessment of AVI and B_7_ in Food Matrices

AVI and B_7_ influence and their content in some food matrices were estimated by the ELISAs based on biotinylated hapten-based coating antigens. Standards of AVI or B_7_ as well as these components in food matrices could inhibit the binding of B_7_–haptens to coated SAV in a dose-dependent manner. Thus, the solutions of standards/samples were added to SAV-coated wells together with B_7_–hapten and incubated for 1 h at 25 °C. After this competition stage, the assay was completed as described above. Standard-generated plots served to assess the content of AVI and B7 in food matrices and their effect on (strept)avidin-based assays.

For testing eggs, homogenates were prepared from the contents of eggs by vigorous stirring with a magnetic stirrer. Several samples were tested for AVI/B_7_ content. Before analysis in the developed ELISA, the homogenates were diluted 100 times with PBST.

Aliquots of milk were centrifuged for 5 min at 10,000 rpm (7500× *g*) to separate and remove milk fat. Then, the samples, diluted 100 times with PBST, were analyzed in ELISAs.

Liver samples from chicken and beef were homogenized using a blender. Portions of the homogenates (1 g) were vigorously stirred in 4 mL PBST. The obtained extracts were centrifuged at 3000 rpm, and the supernatants were diluted 20-fold with the assay buffer.

## 3. Results and Discussion

### 3.1. Assessment of the SAV–Biotinylated Hapten Complexes as Coating Antigens

The optimization of immunoassay parameters is based on choosing the right ratio between antibody and antigen concentrations. A small-molecule analyte immunoassay, generally a competitive assay, involves the conjugated forms of the antigen. The hapten load in the conjugate can be influenced by the coupling ratio between the hapten and the carrier/enzyme, the presence of available functional groups in the latter, and the conditions of conjugation. Since the final hapten load is difficult to predict, the optimization requires the comparison of conjugates with different loads and the selection of the best one [29]. Thus, a comparison of BT/COL coating conjugates prepared from 10- and 30-fold molar excesses of hapten over GEL showed that a lower hapten loading increased the assay sensitivity, which was confirmed by our earlier observations [30,31].

**Figure 2:**
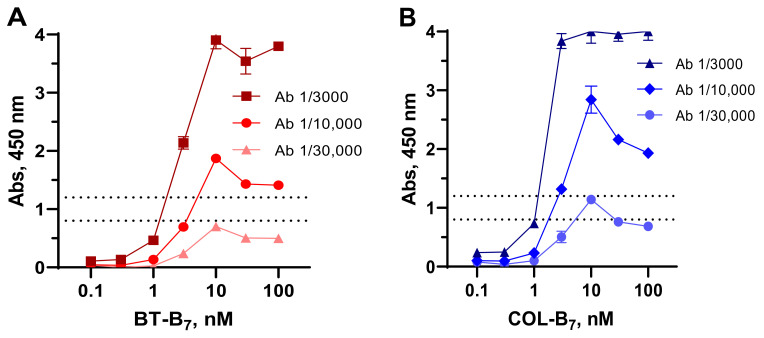
Checkboard titration of BT–B_7_ (**A**) and COL–B_7_ (**B**) on coated streptavidin detected by serially diluted anti-BT (**A**) and anti-PM (**B**) antibodies (Ab) in ELISA. The dashed lines indicate an absorbance range of 0.8–1.2.

As an alternative hapten immobilization approach, a stable complex between SAV coated on the plates and biotinylated hapten was formed. The degree of hapten immobilization on the plates could be controlled more finely by simply titrating B_7_–hapten on the high-affinity binder SAV (Figure 2). The best sensitivity was obtained when using 3 nM solutions of biotinylated haptens and corresponding antibody dilutions, providing an absorbance level of 0.8–1.2.

**Figure 3:**
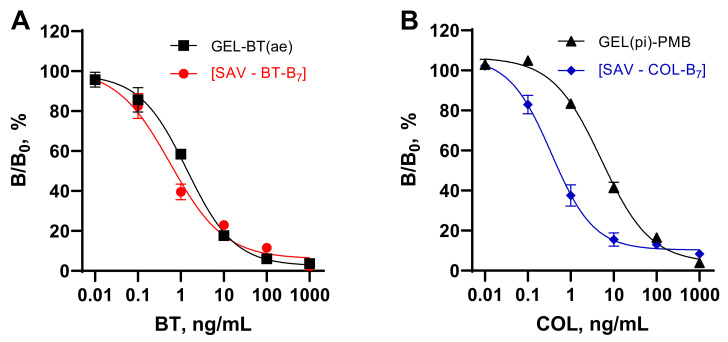
Competitive ELISAs for bacitracin (**A**) and colistin (**B**) based on hapten–protein conjugates (empty symbols) and complexes formed between streptavidin (SAV) and biotinylated (B_7_) hapten (filled symbols).

Thus, two kinds of hapten immobilization, (1) hapten–protein conjugates and (2) coating SAV–hapten–B_7_ complexes were considered in the present study and compared in assay systems for the determination of the peptide antibiotics BT and COL. As shown in Figure 3, the coated complex contributed to a slight increase in the sensitivity of BT determination. The IC_50_ value of SAV–B_7_–BT ELISA was 0.57 vs. that of 1.4 ng/mL of GEL–BT(ae). A more pronounced effect was observed in relation to COL determination.

The IC_50_ value of 5.2 ng/mL achieved in the ELISA based on GEL(pi)–PMB could be improved up to 0.34 ng/mL when using the SAV–B_7_–COL complex. Thus, the SAV-mediated coating of the biotinylated hapten allowed for a finer optimization of the density of the hapten on the plates compared to the hapten load achieved on the conjugate. The orientation of the hapten into the well volume could also contribute to its steric accessibility and better reactivity in comparison with determinants of the conjugate partly hidden as a result of coating. The improved sensitivity of BT and COL determination achieved using this coating technique was comparable to and more often exceeded that in other reports [22,23,24].

### 3.2. Specificity of the Developed SAV–B_7_–BT and SAV–B_7_–COL-Based ELISAs

The specificity of the developed tests for BT and COL was assessed by the cross-reactivity of related analogs, other cyclic peptide antibiotics, and antibiotics of different families, whose concentration is regulated in food (Table 2). Among the examined substances, cross-reactivity was only found for bacitracin complexed with zinc ions (67%) in the anti-BT assay. The other examined substances did not interfere with antibody binding.

SAV–B_7_–COL-coated ELISA, a heterologous hapten-based assay format, also showed higher COL cross-reactivity (100%) compared to immunizing hapten, PMB (22%), as did the coated COL-conjugate heterologous assay format [25]. Colistin MS is a prodrug form with blocked amine groups, so it was poorly recognized by the antibody. However, as a result of hydrolysis in vivo, CMS was converted into COL and could be detected as active drug [32].

### 3.3. Assessment of Extraneous Avidin Influence on the Assay

**Figure 4:**
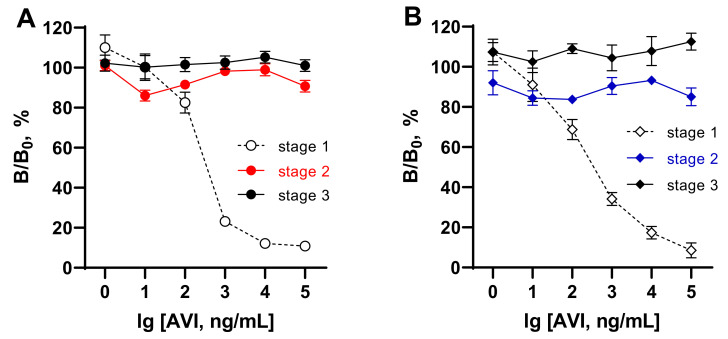
Influence of extraneous AVI on different stages of ELISAs for bacitracin (**A**) and colistin (**B**) based on coated complexes formed between SAV and biotinylated hapten. Complex formation between coated SAV and BT/COL–B_7_ (stage 1), antibody–analyte binding (stage 2), and bound antibody–GAR–HRP interaction (stage 3).

AVI and AVI analogs with biotin-binding activity can be found in the eggs of birds, reptiles, and amphibians [33,34,35]. Bacterial AVI analogs are SAV-isolated from *Streptomyces avidinii* [36], and rhizavidin from the root nodule nitrogen-fixing bacteria *Rhizobium etli* [37]. Tamavidins and Lentiavidins are other representatives derived from edible Tamogitake *(Pleurotus citrinopileatus)* [38] and Shiitake mushrooms (*Lentinula edodes*) [39]. All of these biotin-binding proteins and numerous recombinant products have moderate to extremely high affinity for biotin (Kd = 10^−7^–10^−16^ M); therefore, they can interfere with the “SAV/AVI–B_7_” interaction when present in a sample. Because of this, the effect of extraneous AVI on ELISAs in the detection of BT and COL using the coated SAV–B_7_–hapten complex was examined. This effect was investigated in different stages of the assay. Figure 4 (dashed lines) demonstrates that extraneous AVI (1–100,000 ng/mL) could inhibit the formation of the complexes SAV–BT–B_7_ and SAV–COL–B_7_ in a dose-dependent manner (stage 1). Therefore, the developed assay systems could simultaneously serve as competitive assays for AVI detection. No evident effect at stages 2 and 3 was found when SAV–B_7_ complexes were already formed. Thus, interference from extraneous AVI may be a drawback for assay formats involving “coated antibody–hapten–B_7_” or “coated hapten–antibody–B_7_”, where AVI can interact with unblocked biotin.

### 3.4. Assessment of Extraneous Biotin Influence on the Assay

Mammals cannot synthesize biotin but depend on dietary intake from microbial and plant sources [10]. Nevertheless, B_7_ uptake, accumulation in tissues, especially in the liver, and renal reabsorption of B_7_ determine its presence in animal-derived food. The tissues richest in biotin are chicken and beef liver (0.4–1.9 µg/g), egg yolk (0.3 µg/g), fish (0.05–0.1 µg/g), while the majority of B_7_ in meat is protein-bound [15]. The mentioned B_7_ content is sufficient to cause an undesirable interference in (strept)avidin–biotin-based immunoassay.

**Figure 5:**
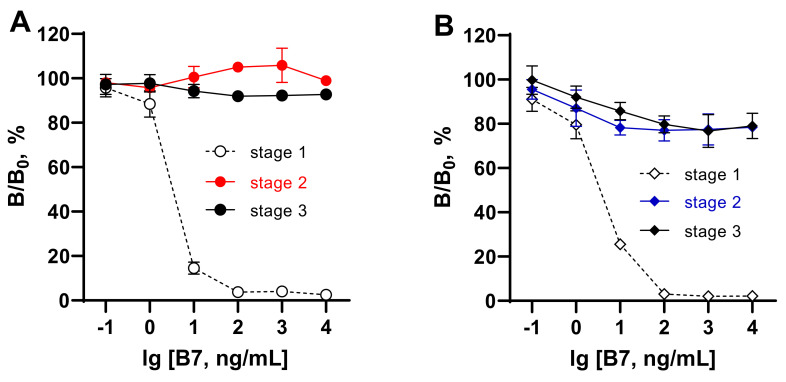
Influence of extraneous biotin (B_7_) in different stages of ELISAs for bacitracin (**A**) and colistin (**B**) based on coated complexes formed between SAV and biotinylated hapten. Complex formation between coated SAV and BT/COL–B_7_ (stage 1), antibody–analyte binding (stage 2), and bound antibody–GAR–HRP interaction (stage 3).

The possible interference from extraneous B_7_ was examined in different stages of the developed assays as reported above for the assessment of AVI influence. As seen in Figure 5, the presence of B_7_ in the test samples up to 10,000 ng/mL (50 μM) could disrupt the formation of the complex between SAV and B_7_ at concentrations >1 ng/mL (Figure 5, stage 1). When the complexes between SAV and B_7_–hapten were already formed in stage 1, the influence of extraneous B_7_ could not strongly affect the binding in the assay ranging between 80 and 110% (Figure 5, stage 2,3).

Thus, these experiments showed that samples with moderate to high AVI or B_7_ content cannot be analyzed using tests in which the analyte is detected during SAV–B_7_ complexation due to the strong influence of endogenous matrix factors. In the assay design considered in this work, the recognition of the analyte by antibodies (stage 2) was separated from the SAV–B_7_ interaction (stage 1) to minimize the possible influence of endogenous AVI/B_7_ from the matrix.

### 3.5. Influence Assessment of Avidin and Biotin Components from Different Matrices

*Egg*. Poultry eggs are real animal-derived samples among possible biotin-binding food matrices, which can be analyzed for antibiotic contaminants such as BT or COL. The maximum concentration of AVI in chicken egg is about 0.05% of the total egg protein (approximately 1800 μg per egg) [33]. B_7_ is also found in eggs, about 7 and 50 µg per 100 g of egg white and yolk, respectively (20.7 µg in a whole egg). Thus, both components of the egg are capable of interfering with the analysis. On the other hand, being components of one sample, they can partly quench each other’s activity. However, their residual inhibitory activity on SAV–B_7_ binding remains unknown. To estimate the inhibitory effect of extraneous AVI/B_7_ from eggs on ELISAs for the detection of BT and COL, homogenates were prepared from eggs produced in poultry farms from seven different country regions (Leningrad Oblast, Nizhny Novgorod Oblast, Yaroslavl Oblast, Tula Oblast, Ryazan Oblast, Udmurtia, and Bashkortostan). Egg homogenates, 100-fold diluted with PBST, could inhibit the binding of the B_7_-labeled haptens to the SAV-coated wells when they were added together with the latter. Using standard curves (dashed lines from Figure 4 and Figure 5), their resulting activity was measured and corresponded to an AVI average content of 90.6 ± 47.5 µg/mL (20.6–145.5) and to a B_7_ average content of 1.98 ± 0.92 µg/mL (0.56–3.0).

*Milk formula*. Milk itself contains negligible amounts of B_7_, about 1 ng/g [15], but some infant formulas are fortified with vitamins. Two infant milk formula, “Agusha-1” and fermented milk formula “Agusha-2”, which were used in recovery experiments of BT/COL, included 2 µg B_7_ per 100 g of product (20 ng/mL), as indicated by the manufacturer.

*Liver.* Animal liver is one of the richest sources of biotin [15]. Samples of chicken and beef liver were examined for their interference in the formation of the SAV–B_7_–hapten complex and demonstrated inhibitory activity equivalent to 325 ± 25 ng/g and 164 ± 36 ng/g, respectively.

Thus, the considered food matrices include a sufficient level of endogenous factors, AVI or B_7_, which can inhibit the binding of SAV- or B_7_-labeled reagents and distort the assay results. This was exemplified by the influence of the matrix at the stage of binding of the biotinylated hapten to the immobilized SAV. However, despite the stage of analyte recognition and the stage of SAV–B7 interaction being separated in the developed assay systems, the influence of endogenous matrix factors on the quantification of analytes was tested using standard BT and COL curves.

### 3.6. Influence of AVI and B_7_ on the Quantification of BT and COL with ELISAs Based on the Coated SAV–B_7_–Hapten Complex

**Figure 6:**
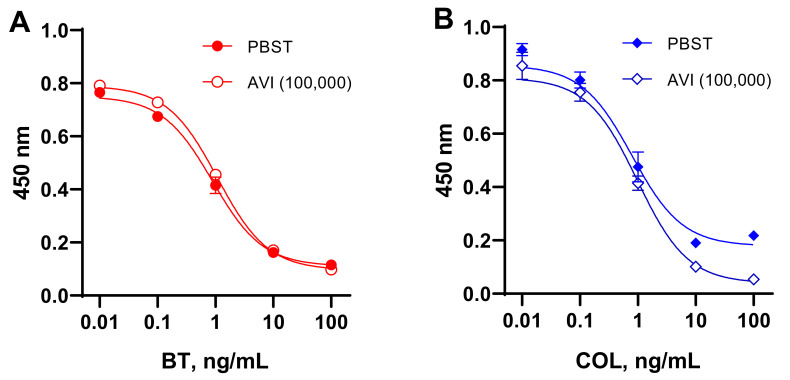
Standard curves for bacitracin (**A**) and colistin (**B**) determination in SAV–B_7_–hapten ELISAs generated in PBST and buffer enriched with avidin (AVI) at 100,000 ng/mL.

**Figure 7:**
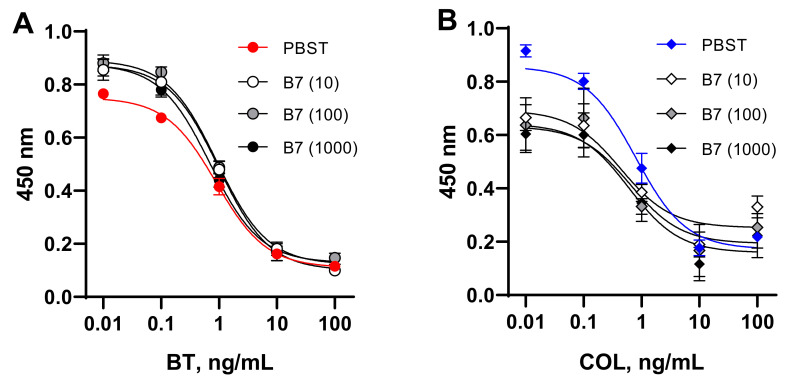
Standard curves for bacitracin (**A**) and colistin (**B**) determination in SAV–B_7-_hapten ELISAs generated in PBST and buffer enriched with biotin (B_7_) at 10, 100, and 1000 ng/mL.

To simulate the effect of AVI and B_7_ contained in a matrix on the quantitative determination of BT and COL, we examined the calibration curves obtained using media enriched with these factors. Analyte standards were prepared in PBST and in buffer with the maximum expected concentration of the studied factors in food samples (AVI, 100,000 ng/mL, B_7_, 10–1000 ng/mL). The comparative study of standard curves generated in buffer and media containing AVI (Figure 6) and B_7_ (Figure 7) revealed a discrepancy between the compared curves. These experiments with model AVI/B_7_-rich food matrices confirmed the interference of these endogenous factors in analyte quantification in (strept)avidin-based assays. This finding provides an explanation for the failure of the recovery of BT and COL from AVI/B_7_-rich foodstuffs using these kinds of assays.

## 4. Conclusions

An alternative approach for hapten coating on plates was realized using the oriented binding of biotinylated BT and COL to immobilized SAV. Such formed complexes and BT/COL conjugated to protein carriers were compared as coating antigens in competitive ELISAs for the determination of these cyclic peptide antibiotics in food matrices. The simple titration of B_7_–haptens in SAV-coated plates provided a finer optimization of coated hapten load compared with hapten load on the conjugates. Using the coating complexes SAV–B_7_–BT and SAV–B_7_–COL instead of the coating conjugates GEL–BT(ae) and GEL(pi)–PMB improved the sensitivity of the determination of BT and COL by 2 and 15 times, achieving 0.6 and 0.3 ng/mL, respectively. The applicability of an immunoassay based on (strept)avidin–biotin interactions for the detection of analytes in food matrices rich in AVI or B_7_ was also challenged in this work. The possible interference of AVI or B_7_ from a matrix in different stages of the immunoassay was investigated. As a result, the developed competitive indirect ELISAs based on coated SAV–B_7_–BT and SAV–B–COL, in addition to determining the main analytes, BT and COL, were applied for assessing AVI and B_7_ content in food matrices such as eggs, infant milk formula enriched with B_7_, and chicken and beef liver. Thus, due to the dose-dependent inhibition of the binding of B_7_–hapten to SAV, these ELISAs proved to be suitable for solving additional analytical tasks, detecting AVI and B_7_ up to 10 and 1 ng/mL, respectively. The interaction between SAV and B_7_–hapten was the one most influenced by extraneous AVI and B_7_. The detection of the analyte by an antibody occurs at different stages of the immunoassay, which minimizes the possible interference from AVI and B_7_ contained in the matrix. However, an adequate quantitative assessment of BT and COL in food matrices rich in AVI/B_7_ was not implemented in the developed (strept)avidin–biotin-based immunoassays. Modification of the standard curves in a matrix-free but AVI/B_7_-enriched environment provided evidence of the influence of these factors on the quantification of the analytes. This explains the failure of numerous recovery experiments with fortified nutritional matrices. Thus, summarizing the experience gained in the course of this study, it is recommended to avoid immunoassays based on avidin–biotin interactions when analyzing biosamples containing these factors.

## Figures and Tables

**Table 1:** Limitations of residual bacitracin (BT) and colistin (COL) content in foodstuff established by the European Union (EU) and the Eurasian Economic Union (EAEU).

Foodstuffs	MRLs (µg/kg) in EU [19]		MRLs (µg/kg) in EAEU [20]	
	BT	COL	BT	COL
Milk	100	50	NE **	50
Eggs	NE	300	<20	300
Meat, Fish, Poultry	150 *	150	<20	150
Liver	150 *	150	<20	150
Kidney	150 *	200	<20	200

* rabbit; ** not established.

**Table 2:** Specificity of the ELISAs developed based on coated biotinylated haptens.

Analyte	Competitive ELISA Based on Coated Complexes			
	SAV–B_7_–BT		SAV–B_7_–COL	
	IC_50_, ng/mL	CR,%	IC_50_, ng/mL	CR,%
Bacitracin A	**0.57**	**100**	>10,000	<0.01
Zn-bacitracin	0.85	67	>10,000	<0.01
Colistin	>10,000	<0.01	**0.34**	**100**
Colistin MS	>10,000	<0.01	9.2	3.7
Polymixin B	>10,000	<0.01	1.5	22.4
Actinomycin D	>10,000	<0.01	>10,000	<0.01
Vancomicin	>10,000	<0.01	>10,000	<0.01
Virginiamycin M1	>10,000	<0.01	>10,000	<0.01
Virginiamycin S1	>10,000	<0.01	>10,000	<0.01
Tetracyclin	>10,000	<0.01	>10,000	<0.01
Erythromycin	>10,000	<0.01	>10,000	<0.01
Tylosin	>10,000	<0.01	>10,000	<0.01
Lasalocid	>10,000	<0.01	>10,000	<0.01
Salinomycin	>10,000	<0.01	>10,000	<0.01
Lincomycin	>10,000	<0.01	>10,000	<0.01
Neomycin	>10,000	<0.01	>10,000	<0.01

The values for the main analyte are highlighted in bold; IC_50_, half-inhibition concentration; CR, cross-reactivity.

## Data Availability

The datasets used and/or analyzed during the current study are available from the corresponding author on request.
